# Therapeutic surfactant-stripped frozen micelles

**DOI:** 10.1038/ncomms11649

**Published:** 2016-05-19

**Authors:** Yumiao Zhang, Wentao Song, Jumin Geng, Upendra Chitgupi, Hande Unsal, Jasmin Federizon, Javid Rzayev, Dinesh K. Sukumaran, Paschalis Alexandridis, Jonathan F. Lovell

**Affiliations:** 1 Department of Biomedical Engineering, University at Buffalo, State University of New York, Buffalo, New York 14260, USA; 2 Department of Chemical and Biological Engineering, University at Buffalo, State University of New York, Buffalo, New York 14260, USA; 3 Department of Chemistry; University at Buffalo, State University of New York, Buffalo, New York 14260, USA

## Abstract

Injectable hydrophobic drugs are typically dissolved in surfactants and non-aqueous solvents which can induce negative side-effects. Alternatives like ‘top-down' fine milling of excipient-free injectable drug suspensions are not yet clinically viable and ‘bottom-up' self-assembled delivery systems usually substitute one solubilizing excipient for another, bringing new issues to consider. Here, we show that Pluronic (Poloxamer) block copolymers are amenable to low-temperature processing to strip away all free and loosely bound surfactant, leaving behind concentrated, kinetically frozen drug micelles containing minimal solubilizing excipient. This approach was validated for phylloquinone, cyclosporine, testosterone undecanoate, cabazitaxel and seven other bioactive molecules, achieving sizes between 45 and 160 nm and drug to solubilizer molar ratios 2–3 orders of magnitude higher than current formulations. Hypertonic saline or co-loaded cargo was found to prevent aggregation in some cases. Use of surfactant-stripped micelles avoided potential risks associated with other injectable formulations. Mechanistic insights are elucidated and therapeutic dose responses are demonstrated.

## Abstract (web summary)

The excipients used to solubilise hydrophobic drugs sometimes interfere with drug behaviour or induce adverse side effects once injected. Here, the authors use a low-temperature process to obtain surfactant-stripped micelles with high drug concentration for delivery of a wide range of hydrophobic cargoes.

## Graphical abstract

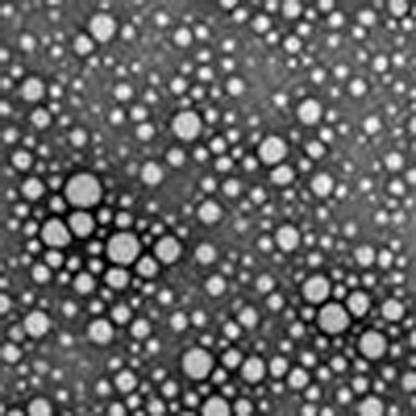

Of over 2,000 approved drugs in 2015 in the DrugBank database^1^, 14% have predicted LogP values greater than 4, corresponding to very hydrophobic compounds which partition over 10,000 times more preferentially in octanol than in water. To administer these compounds by injection, dissolution is required, and water-based solutions avoid the risks of embolisms resulting from injection of oils^2^,^3^,^4^. Depending on the hydrophobicity and chemical properties of the active pharmaceutical ingredient (API), injectable hydrophobic drugs are dissolved with a range of solubilizing excipients^5^. Non-ionic surfactants including Cremophor EL and Tween-80 are used in numerous clinical formulations but modulate drug behaviour and carry the risk of side-effects including anaphylactic shock and neurotoxicity^6^,^7^.

One approach for injection of hydrophobic APIs involves ‘top-down' methods to reduce drug size into sub-micron particles via milling or pressure homogenization^8^. This practice has been successfully applied to orally administered medicines^9^,^10^. However, injectable APIs must be strictly free from microbial contamination and such drug suspensions usually contain particles too large for sterile filtration, a preferred sterilization method. Suspensions tend to settle out of solution, presenting the possibility of inaccurate dosing. Furthermore, administration of very large particles poses the risk of embolism^11^.

Given the challenges facing top-down approaches, there has been interest in the development of nanoscale drug-delivery vehicles formed from bottom-up methods^12^,^13^,^14^. These include nanocrystals^15^, liposomes^16^, albumin^17^, polymer nanoparticles^18^, micelles^19^ and cyclodextrins^20^. Nanocrystals yield the highest ratio of drug to excipient, but face the same impeding challenges as top-down approaches to injectable drug suspensions. Emulsion-templated freeze drying techniques produce dry monoliths that can be smoothly dispersed in liquids for injection, but further research is ongoing to better develop into this methodology^21^,^22^. Liposomal doxorubicin has been shown to reduce cardiotoxicity, although it is not necessarily more efficient than the free drug^23^. Albumin-bound paclitaxel has performed well in clinical trials^24^, but this may be attributed to higher dosing enabled by elimination of the solubilizing surfactant. Thus, clinically viable nanoscale drug-delivery systems have become successful by addressing problems with current clinical formulations, as opposed to becoming a generalized vehicle for hydrophobic drug solubilization, a role that has arguably been assumed by non-ionic surfactants.

Compared with the API itself, the excipient in nanoscale drug-delivery vehicles represents a substantially larger component of the total injected mass, particle size and molarity. Systems that use too little solubilizing excipient generally cannot yield small and stable formulations. Liposomal formulations of doxorubicin can have drug to lipid mass ratios as high as 0.3:1, but are typically closer to 0.1:1 (ref. 25). Polymeric nanoparticles usually have a similar range of drug to polymer mass ratios^26^. A drug-delivery system with a higher drug to excipient ratio could be useful as a generalized vehicle to solubilize hydrophobic drugs. This would minimize side-effects of the vehicle itself and might also enable higher concentrations of drug to be used, which would be useful for situations in which drugs need to be administered in smaller volumes.

Micelles based on block copolymers, another type of surfactant, have been used for drug delivery and some Pluronic (Poloxamer, triblock copolymers of poly(ethylene oxide)-poly(propylene oxide)-poly(ethylene oxide) (PEO-PPO-PEO)), are approved excipients for pharmaceutical use^27^,^28^,^29^. Pluronics have shown promise due to their high drug loading and self-assembly properties^30^. They are commercially available at large scale, with varying poly(ethylene oxide) and poly(propylene oxide) block lengths and ratios, so that surfactant parameters can easily be varied^31^,^32^.

In this study, Pluronic is used together with hydrophobic drugs to develop surfactant-stripped induced frozen micelles (ss-InFroMs) as a new class of drug-delivery vehicles suitable for delivering a wide range of hydrophobic cargo. By lowering the temperature to modulate the critical micelle concentration of Pluronic, loose and free surfactant can be stripped away, leaving behind concentrated drug frozen micelles with extremely high drug-to-excipient ratios.

## Results

### Surfactant-stripped induced frozen micelles (ss-InFroMs)

**Figure 1:**
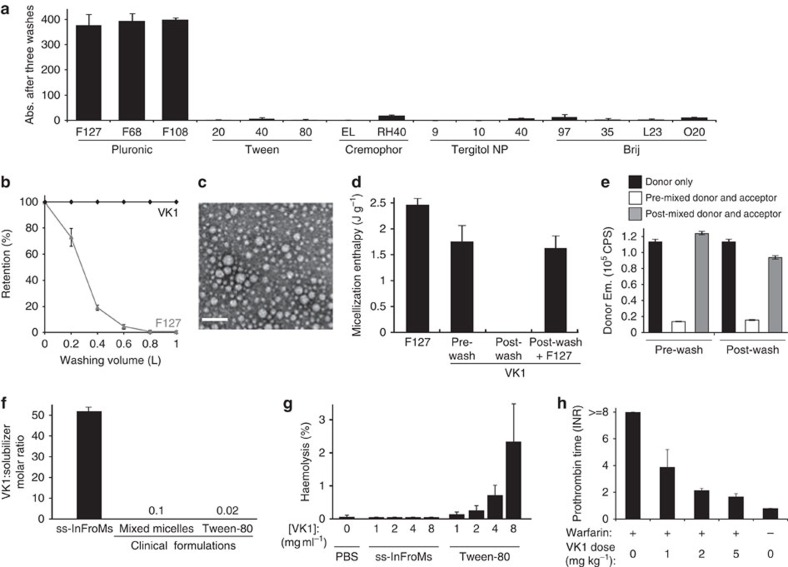
Surfactant-stripped induced frozen micelles (ss-InFroMs). (**a**) Retentate absorbance of a hydrophobic dye, ONc, following dissolution in indicated surfactants (10% w/v) and repeated centrifugal filtration at 4 °C. (**b**) Retention of Vitamin K1 (VK1) and F127 during surfactant stripping by dialfiltration at 4 °C. Values are based on recovery of compounds in the filtrate. (**c**) Negative-stained transmission electron micrographs of VK1 ss-InFroMs. Scale bar, 200 nm. (**d**) Micellization enthalpy of F127, pre wash VK1 InFroMs, and VK1 ss-InFroMs measured in water or in F127, determined by differential scanning calorimetry. (**e**) Exchange of a hydrophobic FRET dye pair co-loaded in InFroMs with VK1. (**f**) Molar ratio of drug to solubilizing excipient for VK1. (**g**) Haemolysis of human erythrocytes by a phosphate buffered saline (PBS) control or by the indicated VK1 formulations. (**h**) Blood clotting in mice exposed to warfarin for 24 h and intravenously administered VK1 ss-InFroMs. Values show mean ±s.d. for *n*=3, or *n*=4–5 for haemolysis analysis and animal studies.

Pluronic F127 (F127) has a concentration-sensitive critical micellar temperature (CMT)^33^. The approach of modulating temperatures above and below Pluronic CMT was used to reversibly and thermochromically switch near infrared (NIR) dyes from nanoparticulate to microcrystalline form^34^. We found that a hydrophobic NIR dye, 5,9,14,18,23,27,32,36-octabutoxy-2,3-naphthalocyanine (ONc), could form induced frozen micelles in aqueous solution using F127 (refs 35, 36). When the temperature was lowered below the CMT, free and loose surfactant (without incorporated ONc) converted to unimeric form and could be removed by membrane filtration, leaving pure and highly concentrated frozen micelles. To determine if this phenomenon was unique to Pluronics, ONc was solubilized with other common surfactants (10% w/v aqueous solutions) including Tween, Cremophor, Tergitol, and Brij. 10% Pluronic corresponds to a concentration above the CMC but avoids formation of mesophase structures that would induce additional complexity in the self-assembly process (see Supplementary Table 1). When subjected to low temperature centrifugal filtration, only Pluronic enabled free surfactant removal, yet retention and concentration of the cargo (Fig. 1a). The other surfactants were not suitable likely because they could not induce the formation of kinetically frozen micelles or because they were not amenable to CMC-switching for low-temperature surfactant removal and the resulting increase in cargo concentration.

Having established Pluronic as possibly the only broadly-available biocompatible surfactant suitable for generating ss-InFroMs, we examined if these could also be generated from therapeutic molecules. Phylloquinone (vitamin K_1_, VK1) is administered clinically to both neonates and warfarin-overdosed patients to prevent or correct deficient blood clotting and has a similar hydrophobicity as ONc (predicted LogP of 8.5 for VK1 and 9.3 for ONc). Following dissolution in a 10% w/v F127 aqueous solution, the temperature of the VK1 solution was lowered to 4 °C, and the vast majority of F127 could be selectively stripped away using diafiltration, leaving behind pure and concentrated VK1 ss-InFroMs (Fig. 1b). Transmission electron microscopy showed that ss-InFroMs formed 70 nm diameter spherical particles (Fig. 1c), consistent with dynamic light scattering (DLS), which showed that no significant size change occurred during storage (Supplementary Fig. 1). On the basis of our recently reported geometric calculation estimates^35^, each 70 nm particle contained ∼1.8 × 10^5^ VK1 molecules. Powder diffraction analysis of freeze-dried ss-InFroMs did not reveal any crystallinity, suggesting an unorganized stacking of VK1 in hydrophobic nano-phased pockets within the ss-InFroMs (Supplementary Fig. 2).

Differential scanning calorimetry (DSC) was used to probe the interactions between hydrophobic cargo and F127. Compared with a 10% F127 aqueous solution containing pristine micelles, the addition of VK1 to a 10% F127 solution resulted in a 30% decrease in micellization enthalpy during heating and cooling cycles (Fig. 1d and Supplementary Fig. 3). Thus, the majority of the F127 behaved thermodynamically similarly to free micelles following VK1 addition. As expected, following surfactant stripping, ss-InFroMs exhibited no detectable micellization energy change, since all free F127 was removed and the micelles were kinetically frozen. When purified ss-InFroMs were diluted back into a 10% F127 solution, micellization energy returned to characteristic levels of the pre-washed VK1 samples. This suggests that ss-InFroMs attract some free surfactant as a loosely bound F127 layer surrounding a core of tightly bound F127 and VK1. The DSC cooling curves (corresponding to unimerization) were broader than the heating curves (corresponding to micellization), whereas F127 alone exhibited fully reversible heat flow (Supplementary Fig. 3). This can be explained by the equilibrium between loosely bound F127 and free F127, which makes it possible to remove the loosely bound layer. A schematic process of the proposed CMC switching and washing process is illustrated in Supplementary Fig. 4.

A Forster resonance energy transfer (FRET) VK1 reporter system was developed to study hydrophobic cargo exchange using the donor dye 2,9,16,23-tetra-tert-butyl-29H-31H-phthalocyanine (BPc), which has similar hydrophobicity to VK1. When solubilized alone with F127, BPc was non-fluorescent. However, when loaded at 1% together with 99% VK1, BPc became highly fluorescent with altered spectral properties, due to reduction in homoaggregation-induced quenching (Supplementary Fig. 5a,b). BPc-doped VK1 InFroMs (since these particular micelles were not stripped of surfactant, we refer to them as InFroMs, as opposed to ss-InFroMs) were used as a donor to VK1 InFroMs doped with a FRET acceptor, Zinc-2,11,20,29-tetra-tert-butyl-2,3-naphthalocyanine (ZnBNc), another hydrophobic dye which has good absorption spectral overlap with BPc emission (Supplementary Fig. 5c). When donor and acceptor dyes were pre-mixed by dissolving them with VK1 in a single methylene chloride (DCM) solution followed by dropwise addition into F127 to generate InFroMs, strong FRET was observed as evidenced by an 80% decrease in donor emission (Fig. 1e). However, when donor and acceptor micelles were combined after formation, no appreciable energy transfer occurred. Even following surfactant stripping and 2 weeks later, the same lack of cargo exchange was observed, demonstrating a strongly kinetically frozen nature of InFroMs (Fig. 1e and Supplementary Fig. 5d,e).

NMR experiments were conducted to gain insights on how InFroMs change following surfactant-stripping. The diffusion coefficients of VK1 protons within InFroMs were ∼3 orders of magnitude lower than those of freely rotating small molecules (typically in the range of 10^−9^ m^2^ s^−1^), as expected for drugs incorporated in micelles (Supplementary Fig. 6a, 6b). Following surfactant stripping, the viscosity-corrected diffusion coefficient of VK1 decreased further (Supplementary Fig. 6a, 6b), as removal of excess F127 enabled drugs to become more locked within micelles. In the pre-wash sample, additional peaks besides those characteristic of F127 and VK1 were observed. Small peaks at 8.1, 6.4, 6.0, 5.2 and 4.2 p.p.m. in the pre-wash VK1 sample were observed which may originate from a small proportion of VK1 only loosely bound to F127. The diffusion coefficients of those smaller peaks were greater than the diffusion coefficients of the major peaks of VK1, presumably tightly bound to F127. The small peaks likely correspond to small proportion of more rapidly diffusing, peripherally bound VK1 in equilibrium with VK1 in the core of the micelle. These peaks disappeared after surfactant-stripping, suggesting that the loosely bound VK1 was driven deeply into the frozen micelles as excess surfactant was removed (Supplementary Fig. 6b). Correlation spectroscopy (COSY) conclusively confirmed that the small peaks observed were from VK1, and not other impurities (Supplementary Fig. 7). On the basis of NMR peak analysis, diafiltration-based surfactant stripping resulted in a final drug to solubilizer (VK1:F127) molar ratio of over 50:1 (Fig. 1f and Supplementary Fig. 8). This is orders of magnitude greater than mixed micelle and Tween-80 formulations of VK1 that have been used clinically. VK1 ss-InFroMs could also be generated with Pluronic F68 in a similar manner (Supplementary Fig. 9a), achieving a VK1:F68 molar ratio of 25 (Supplementary Fig. 9b). Powder diffraction analysis did not reveal any crystallinity in F68 VK1 ss-InFroMs, suggesting VK1 was dispersed similarly to F127 ss-InFroMs (Supplementary Fig. 9c). The removal of excess surfactant not only enabled high drug to surfactant ratios, but also enabled VK1 concentration to unusually high levels (150 mg ml^−1^, see Table 1). This could be potentially be useful for applications where limited injection volumes are preferred. The large excess of F127 used before surfactant stripping was found to be essential, since when VK1 was simply directly dissolved with the same molar ratio of 50:1 VK1:F127 and sonicated, drug solubilization was ineffective (Supplementary Fig. 10). Having a concentrated drug solution could be useful for administration of low volumes of API. We found that the surfactant Tween-80 could also dissolve VK1 at a high concentration (100 mg ml^−1^). The haemolytic activity of these two solubilization systems was examined in fresh human red blood cells to examine side effects of a potential high API concentration, low injection volume approach. As shown in Fig. 1g, no appreciable human erythrocyte damage was induced by VK1 ss-InFroMs whereas the Tween-80 induced haemolysis, due to surfactant action of lysing membranes.

VK1 ss-InFroMs were assessed for countering the effects of warfarin in mice. Following intravenous administration, VK1 ss-InFroMs exhibited a circulating half-life of 6.7 h (Supplementary Fig. 11), showing that they were not rapidly cleared from the blood, as would be the case for pure drug suspensions with large particle size. Next, mice were exposed to warfarin for 24 h and then were administered VK1 ss-InFroMs via intravenous injection. As shown in Fig. 1h, VK1 ss-InFroMs rescued mice from warfarin-induced blood clotting inhibition in a dose-dependent manner, with efficacy in the same general range as previous reports using conventionally solubilized VK1 (ref. 37). Thus, in mice, VK1 ss-InFroMs became bioavailable *in vivo* and provided good efficacy without observable adverse side-effects.

### Hypertonic saline to enhance generation of ss-InFroMs

**Figure 2:**
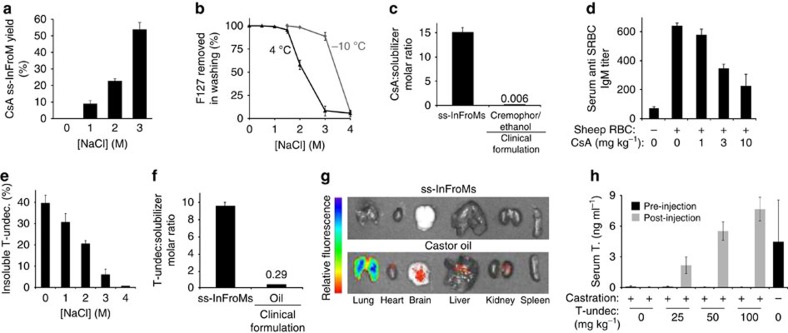
Hypertonic saline enhancement of ss-InFroM formation. (**a**) Cyclosporine A (CsA) ss-InFroM yield formed at varying salt concentrations. (**b**) Lower washing temperatures enable surfactant stripping in hypertonic saline. (**c**) Molar ratio of drug to solubilizing excipient for CsA. (**d**) Immunosuppresion induced by CsA ss-InFroMs, based on IgM response to intraperitoneal injection of sheep red blood cells. (**e**) Hypertonic saline effect on the loading of testosterone undecanoate (T-undec) in F127 micelles. (**f**) Molar ratio of drug to solubilizing excipient for T-undec. Values show mean ±s.d. for *n*=3, or *n*=4–5 for animal studies. (**g**) Fluorescence images of extracted organs following intravenous injection of T-undec co-loaded with 2% BPc in ss-InFroMs or castor oil. Representative of 3 independent experiments. (**h**) Testosterone levels in castrated mice before and 1 week following subcutaneous injection of indicated T-undec doses in ss-InFroM form. Testosterone (T.) levels were assessed by ELISA specific for active testosterone (*n*=3 mice per group).

Whereas VK1 and ivermectin, an antiparasitic drug, readily formed ss-InFroMs in water with F127 (see Table 1), initial experimentation with other drugs such as cyclosporine A (CsA) did not yield positive results. CsA is an immunosuppressive cyclic peptide commonly administered orally and intravenously used to prevent transplant rejection. Ivermectin and CsA have similar LogP values of 4.4 and 4.1, respectively, both lower than VK1, which has a LogP value of 8.5. Thus, due to other chemical properties, ivermectin, but not CsA could partition favorably into F127 micelles and avoid aggregation during surfactant stripping in deionized water. To overcome this problem for CsA, hypertonic saline was investigated as a means to improve micelle stability. With addition of 3 M sodium chloride, CsA ss-InFroMs were able to form stable nanoparticles following surfactant-stripping (Fig. 2a). Although this is a high concentration of salt, current intravenous drug administration usually involves dilution of a drug concentrate before administration, so using this approach would convert the hypertonic saline to physiological levels. Presumably, the salt makes the solution more polar, resulting in more stable partitioning of the hydrophobic cargo into the frozen micelle core. Not only does salt impact micellization^38^, it also lowers the CMC, thereby making the surfactant F127 stripping process from CsA ineffective at 4 °C (Fig. 2b). However, since salt also depresses the freezing point of water, subzero temperatures could be used to remove F127 from CsA. The resulting CsA ss-InFroMs had a diameter of ∼100 nm and were stable for over 2 weeks, based on DLS measurement (Supplementary Fig. 12). Like VK1, the CsA molar ratio in ss-InFroMs was orders of magnitude higher than existing clinical formulations (Fig. 2c). Interestingly, Pluronic F68 could not form InFroMs even with hypertonic saline (Supplementary Fig. 13). This might be due to the substantially decreased hydrophobic PPO block length (<50%), and an order of magnitude higher CMC compared with F127 (Supplementary Table 1), leading to weaker micellar hydrophobic interactions that did not sufficiently drive association of the drug with the Pluronic F68. When CsA ss-InFroMs were administered to mice before injection of immunogenic sheep red blood cells, they safely and effectively inhibited the immune system IgM response in a dose dependent manner, as expected for an immunosuppressive drug (Fig. 2d).

Saline loading for another hydrophobic drug, testosterone undecanoate (T-undec) was investigated. T-undec is used for treatment of hypogonadism as an intramuscularly-injected oil formulation. As shown in Supplementary Fig. 14, hypertonic saline generated T-Undec. F127 solutions that were clear, showing the drug was driven into F127 micelles of relatively small size. Following centrifugation, negligible unincorporated, insoluble drug was detected when highly hypertonic saline was used (Fig. 2e). To strip excess F127 from T-undec InFroMs in 4 M sodium chloride, diafiltration was conducted at −16 °C using a custom-built propylene glycol and dry ice submersion bath. Excess F127 was removed effectively (over 85% of the starting F127) by low temperature washing, resulting in T-undec ss-InFroMs, with a size of ∼110 nm (Supplementary Fig. 15). The T-undec to excipient molar ratio in ss-InFroMs was over 30 times higher considering that the oil used in current formulation is an excipient itself (Fig. 2f). Oil is commonly and safely used for intramuscular administration of steroidal compounds. However, microembolisms can occur with accidental injection into veins or translocation of oil into the circulatory system. To evaluate the capability of ss-InFroMs to bypass this issue, T-undec ss-InFroMs were formed with 2% fluorescent BPc doped as a detection probe, as that amount of BPc yielded the highest fluorescence signal (Supplementary Fig. 16). Shortly following intravenous injection, no significant fluorescence was detected from extracted organs (Fig. 2g) as most of the ss-InFroMs remained in circulation (Supplementary Fig. 17). In contrast, the castor oil formulation using the same amount of BPc demonstrated accumulation in the lungs, likely due to the formation of microemboli. The high injectable drug concentrations achieved with ss-InFroMs could enable new parenteral dosing routes for hydrophobic compounds that have been limited largely to intramuscularly administration in oil. Castrated mice that were treated subcutaneously with T-undec ss-InFroMs exhibited a dose response in their testosterone levels which could reach similar levels to non-castrated mice (Fig. 2h).

### Cargo co-loading to enhance generation of ss-InFroMs

**Figure 3:**
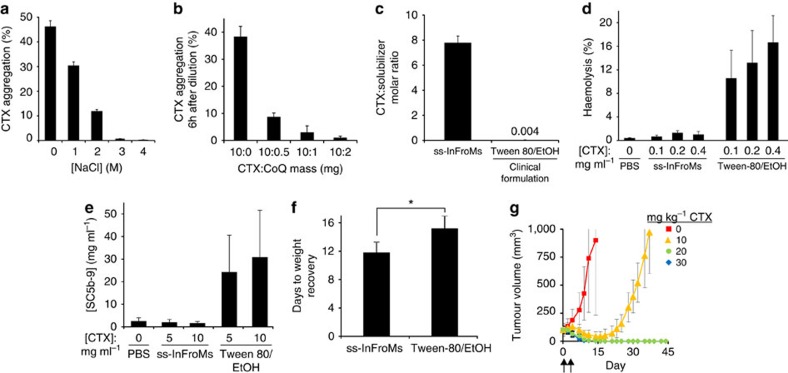
Cargo co-loading to enhance stability of ss-InFroMs. (**a**) Hypertonic saline enhanced the yield of cabazitaxel (CTX) InFroMs. (**b**) Coenzyme Q10 (CoQ) co-loading improved CTX InFroM stability following dilution into water. (**c**) Molar ratio of drug to solubilizing excipient for CTX formulations. (**d**) Haemolysis of human erythrocytes induced by CTX formulations. (**e**) Complement activation in human plasma induced by CTX formulations. (**f**) Time needed for mouse weight recovery after two injections of 30 mg kg^−1^ CTX on days 0 and 4. Mice recovered significantly faster with ss-InFroMs (Student's unpaired *T*-test, *P*<0.05) (**g**) Anti-tumour efficacy of CTX ss-InFroMs intravenously injected in nude mice bearing subcutaneous MIA Paca-2 tumours. Two injections were given with the indicated doses on days 0 and 4 (indicated by arrows). Values show mean±s.d. for *n*=3; or *n*=5–6 for animal studies; or *n*=4 for blood tests.

The taxane family is used as frontline chemotherapy for numerous cancer indications and there is interest in developing novel and stable taxane formulations^39^. As taxanes are administered intravenously in rather large doses (that is, 100 s of mgs), high amounts of Cremophor or Tween-80 and ethanol (up to 25 ml each) are usually used to dissolve the drug and are co-administered to patients. Initial attempts at dissolving taxanes in F127, even in hypertonic saline, yielded formulations that were prone to aggregation. To overcome this problem, we developed a hypertonic saline strategy together with cargo co-loading to generate ss-InFroMs formed from cabazitaxel (CTX), a third generation taxane. At 10mgmL^−1^, CTX could not be solubilized in F127 without hypertonic saline (Fig. 3a). However, since hypertonic saline must be diluted before intravenous administration, an additional obstacle encountered was that, following dilution in plain water, CTX rapidly aggregated. We hypothesized that addition of a co-loaded cargo might slow or prevent this. Coenzyme Q10 (CoQ), a naturally occurring and abundant hydrophobic vitamin found in the mitochondria inner membrane, was co-loaded with CTX in F127 micelles. As shown in Fig. 3b, with a CTX:CoQ mass ratio of 10:2, no appreciable precipitation of CTX occurred in 6 h following dilution in water, whereas without CoQ co-loading, 40% of the CTX precipitated. This period would be sufficient to administer the drug via infusion to the patient. The kinetics of precipitation are shown in Supplementary Fig. 18. Within InFroMs, CoQ likely interferes with those intermolecular CTX interactions which lead to aggregation. The salt-assisted co-loading method was also applied to other taxanes. CoQ and another non-toxic co-loading vitamin, α-tocopherol (vitamin E), were effective for generating stable docetaxel (Supplementary Fig. 19) and paclitaxel (Supplementary Fig. 20) InFroMs.

Low-temperature CMC switching washing was used to generate CTX ss-InFroMs, with a size of 60 nm (Supplementary Fig. 21). As with the other ss-InFroMs described, the drug to solubilizing excipient ratio of CTX ss-InFroMs was orders of magnitude larger than the current clinical formulation, which is based on Tween-80 and ethanol. To assess the toxicity of ss-InFroMs for *in vivo* application, haemolytic activity, complement activation and maximum dose tolerances were investigated. As shown in Fig. 3d, no human erythrocyte damage was induced by CTX ss-InFroMs, whereas haemolysis was induced by a formulation resembling a current clinical formulation based on Tween-80. Figure 3e shows that when incubated in human plasma, no appreciable complement activation product (SC5b-9) was generated by ss-InFroMs whereas the Tween-80 formulation promoted complement activation, as has been reported previously^40^. As shown in Fig. 3f, when CTX was formulated in ss-InFroMs or Tween-80 and was administered with two intravenous injections on days 0 and 4, mice treated with the Tween-80 formulation needed more days to recover lost weight. The efficacy of CTX ss-InFroMs was evaluated in nude mice bearing subcutaneous MIA Paca-2 tumours, which have previously been demonstrated to be sensitive to CTX^41^. As shown in Fig. 3g, 10 mg kg^−1^ dosing effectively delayed tumour growth and 20 and 30 mg kg^−1^ dosing shrank tumours to being undetectable, without regrowth throughout 45 days. ss-InFroMs induced less haemolysis, hypersensitivity and systemic toxicity compared with the Tween-80 formulation, but further studies are required to establish maximum tolerated dose and efficacy of ss-InFroMs, as a carrier that enables higher dosing has been shown to be viable strategy for taxanes in the case of albumin-bound paclitaxel^42^.

### ss-InFroMs formed with diverse hydrophobic drugs

To further validate ss-InFroMs as a generalized platform for formulating a wide range of hydrophobic APIs with minimal solubilizing excipient, we applied surfactant stripping approaches to other bioactive molecules including α-Tocopherol, CoQ, cholecalciferal, ergocalciferol, ivermectin, retinol palmitate and squalene. Hydrophobic cargo was dissolved in Pluronic and hypertonic saline was added as required to generate a clear micelle solution of 10 mg ml^−1^ in 10% F127 (Table 1). After CMC switching and surfactant stripping, molar ratios of drug to F127 ranged from 10 to 55, demonstrating a strikingly high proportion of API in the carrier system. Achievable drug concentration in ss-InFroMs ranged from 7 to 150 mg ml^−1^, with a typical value of ∼50. This drug concentration exceeds typical drug concentrates dissolved in pure surfactants or organic solvent that are diluted before injection. Before measurement, samples were centrifuged at 10,000*g* and no visible aggregates were observed, implying the high concentrations were free of drug aggregates. The small size of ss-InFroMs (<200 nm diameter, with a typical value close to 100 nm), with relatively narrow size distribution (average polydispersity index of 0.2) and theoretical amenability to freeze drying imply they are suitable for larger-scale manufacturing and sterile filtration and storage processes.

## Discussion

Pluronic block copolymers, but not other surfactants, were able to generate induced frozen micelles with sufficiently hydrophobic cargo that could be subjected to CMC-switching and surfactant stripping. This approach enables unusually high drug to excipient molar ratios and drug concentrations, with the hydrophobic cargo remaining in a stable core with minimal inter-micellar exchange. Minimizing the amount of excipient not only holds potential to enable higher dosing due to reduced adverse side-effects but also minimizes unexpected carrier effects that can modulate drug function. The high concentration of drugs achievable with ss-InFroMs could be useful for specific applications where low volumes are desired, such as topical drug delivery.

In several cases, ss-InFroM formation could be assisted with hypertonic saline and hydrophobic cargo co-loading. The chemical properties which determine whether or not APIs will or will not be suitable for ss-InFroM formation have yet to be determined, although less hydrophobic compounds (for example, LogP<3) have not yet been found that are suitable. Further work is also required to characterize the long term stability of ss-InFroMs and to see if they are amenable to lyophilization. Taken together, the data presented here show that ss-InFroMs represent a safe, accessible and generalizable material for formulating hydrophobic APIs with minimal excipient.

## Methods

### Materials

Materials were obtained from Sigma unless otherwise noted. The following were used: Pluronic F127 (Sigma # P2443), Pluronic F108 (Sigma # 542342), Pluronic F68 (Sigma # 412325), Polysorbate 20 (Amresco # M147-1L), Polysorbate 40 (Sigma # 1547936), Polysorbate 80 (VWR # EM-9490), Cremophor EL (Sigma # C5135), Cremophor RH40 (Sigma # 07076), Tergitol NP 9 (Sigma # NP9), Tergitol NP 10 (Sigma # NP10), Tergitol NP 40 (Sigma # NP40S), Brij 97 (Spectrum # B1679), Brij 35 (Spectrum # BR105), Brij L23 (Sigma # 16005), Brij O20 (Sigma # 436240), Vitamin K1 (VK1, VWR # AAAL10575-03), cyclosporine A (CsA, VWR # 89156-334), Testosterone undecanote (T-undec, Matrix # 099258), cholecalciferol (VWR, TCC0314), Retinol Palmitate (VWR # IC15652125), coenzyme Q10 (Sigma # 45-C9538), Docetaxel (LC Labs # D-1000), Paclitaxel (LC Labs # P-9600), Cabazitaxel (CTX, Proactive Molecular Research), Squalene (VWR # AAB20944-22), 5,9,14,18,23,27,32,36-Octabutoxy-2,3-naphthalocyanine (ONc, Sigma # 412074) 2,9,16,23-Tetra-tert-butyl-29H,31H-phthalocyanine (BPc, Sigma # 423157), Zinc 2,11,20,29-tetra-tert-butyl-2,3-naphthalocyanine (ZnBNc, Sigma # 432210), Castor oil (Sigma # 259853), methylene chloride (Fisher), Ethanol (Decon), ethylene glycol (VWR # 1071401), and sodium chloride (Macron # 7532-06).

### Hydrophobic dye dissolution and centrifugal filtration

Three ml of 0.5 mg ml^−1^ ONc in methylene chloride (DCM) was added dropwise to 15 ml 10% (w/v) of various surfactant solutions including Pluronic F127, F108, F68, Cremophor EL, Cremophor RH40, Tween 20, 40, and 80, Tergitol NP 9, 10, 40, and Brij 97, 35, L23, O20. The solutions were stirred for 3 h, centrifuged at 3,500*g* for 10 min and the supernatants were subjected to centrifugal filtration at 4 °C for 75 min. Water was added back to the concentrates and the washing procedure was conducted at 4 °C three times. The absorbance of the resulting supernatant was measured at 860 nm (the absorbance peak of the dye) and values are presented multiplied by the dilution factor used during absorbance measurement.

### Drug solublization and surfactant stripping

In general, 100 mg drug was dissolved in 1 ml DCM and added to 10 ml 10% (w/v) F127 (with or without NaCl as indicated) and stirred until the organic solvent evaporated (typically 3 h). To remove excess surfactant, two methods were used: (1) centrifugal filtration: Solutions were subjected to centrifugal filtration at 3,500*g* with conical filtration devices (100,000 Da molecular weight cutoff; Fisher # UCF9-100-24) at low temperatures (0 °C, 4 °C or −10 °C), until ∼200 μl of concentrate was retained (or the volume of concentrate was unchanged). Water (or an NaCl solution) was added back to the concentrate and the washing procedure was repeated three times. (*2)* peristaltic filtration: For larger scale (>15 ml) or high salt (>2 or 3 M) solutions, surfactant stripping was conducted by diafiltration (Sartorius Vivaflow, 1501008VS) with a peristalsic pump (Masterflex L/S) and tubing (Masterflex 6434-16) at low temperatures (−7 °C for 2 M NaCl, −12 °C for 3 M NaCl and −16 °C for 4 M NaCl). To reach lower temperatures to enable F127 stripping, membrane modules, tubing, and the washing solution were submersed in a mixture of ethylene glycol and ethanol (vol/vol=9:1), and dry ice was used as a cooling agent. Samples were then typically filtered through a 0.2 μm syringe filter unless noted otherwise.

For VK1 ss-InFroMs, 100 mg drug was dissolved in 1 ml DCM and added to 10 ml 10% F127 (w/v) and stirred until the DCM evaporated. To remove unincorporated F127, centrifugal filtration was conducted at 4 °C. The retentate was filtered by a 0.45 μm filter (VWR, 28145-489). For larger scale diafiltration studies, 150 mg VK1 was dissolved 1.5 ml DCM and added to 15 ml 10% F127 (w/v) and stirred until the organic solvent evaporated. The solution was diluted with water to 75 ml and subjected to diafiltration (Sartorius Vivaflow # 1501008VS) to remove unincorporated F127 and 5 fractions (200 ml each) of filtrate were collected for drug and F127 quantification. For direct sonication of VK1, VK1 directly was dissolved in an aqueous F127 solution with the same VK1 to F127 ratio of ss-InFroMs and sonicated for 2 h

For CsA ss-InFroMs, 100 mg drug was dissolved in 1 ml DCM and added to 10 ml 10% (w/v) F127 containing 3 M NaCl and stirred until the organic solvent evaporated. To remove unincorporated F127, peristaltic filtration was conducted at −12 °C with a 3 M NaCl solution as the diafiltration solution. The retentate was filtered by paper (Whatman # 1001-055) and further filtered with a 0.45 μm syringe filter (VWR # 28145-489).

For T-undec ss-InFroMs, 100 mg drug was dissolved in 1 ml DCM and added to 10 ml 10% (w/v) F127 with 4 M NaCl and was stirred until the organic solvent evaporated. To remove unincorporated F127, peristaltic filtration was conducted at −16 °C and 4 M NaCl was used as a diafiltration solution. The retentate was filtered with a 0.45 μm syringe filter.

For CTX ss-InFroMs, 100 mg drug and 20 mg Coenzyme Q10 were dissolved in 1 ml DCM and added to 10 ml 10% (w/v) F127 containing 3.5 M NaCl and stirred until the organic solvent evaporated. To remove unincorporated F127, the peristaltic filtration method was conducted at −14 °C with 3.5 M NaCl used as the diafiltration solution.

### Physical and optical properties

Absorbance was measured with a Lambda 35 UV/VIS or a Lambda XLS spectrophotometer (Perkin Elmer) using cuvettes with 1 cm path length. Differential scanning calorimetry was performed with a TA DSC Q200 instrument. A total of 10–20 mg of 10%(w/v) empty or drug-loaded aqueous surfactant solutions were placed in hermetic aluminum pans. Three consecutive scans were performed between 0–60 °C at a heating/cooling rate of 1 °C/min by using an empty pan as a reference. The pre-wash sample had 5 mg VK1 and 95 mg F127 per gram of sample whereas the F127 control had 100 mg F127 per gram sample, equivalent to 10% (w/v) F127. The post-wash sample had the F127 concentration adjusted to 10% (w/v). Transmission electron microscopy was performed by using a JEM-2010 electron microscope with 1% uranyl acetate staining. Size measurements were carried out with dynamic light scattering using a NanoBrook 90 plus PALS instrument (Brookhaven Instruments). X-ray diffraction powder pattern was measured with freeze dried samples on a Rigaku Ultima IV with operating conditions of 40 KV, 44 mA, and 1.76 kW. The source of the diffractometer used was a Cu K α radiation at a 1.54 Å wavelength with a monochromator filter and analysed in Θ/2Θ mode at room temperature. The 2Θ scan data were collected at a 0.030 interval and the scan speed was 0.5° min^−1^. The technique used for measuring intensities was the focusing beam method. Log *P* values were evaluated using the ALOGPS 2.1 algorithm. For the additional drugs in Table 1, the surfactant stripping process was conducted as indicated above. To concentrate ss-InFroMs, samples were subjected to centrifugal filtration at 3,500*g* for 2 h or until no more filtrate could be removed. Concentrated sample were subjected to centrifugation at 10,000*g* for 5 min and no noticeable pellet or debris was observed. The 2D COSY and NOESY spectra were acquired in D_2_O on a Bruker AMX 600 MHz spectrometer and DOSY was acquired in D_2_O on a Varian Inova 500 MHz at room temperature. A mixing time of 100 ms was used. Viscosity was measured by cannon-fenske routine type viscometers. Viscosity for pre-wash sample was 10.7 cp, whereas post-wash sample was 0.98 cp, and these values were taken into consideration for diffusion coefficient calculations.

### Forster resonance energy transfer

2,9,16,23-tetra-tert-butyl-29H, 31H,phthalocyaine (BPc) and Zinc, 2,11,20,29-tetra-tert-butyl-2,3-naphthalocyanine (ZnBNc) were used as a novel fluorescence donor and acceptor pair. A donor solution was made by dissolving 0.5 mg BPc, 49.5 mg VK1 in 500 μl DCM. An acceptor solution was made by dissolving 5 mg ZnBNc and 45 mg VK1 in 500 μl DCM. A pre-mixed donor and acceptor organic solution was made by combining 0.5 mg BPc, 5 mg ZnBNc and 44.5 mg VK1 in 500 μl DCM. The above three DCM solutions were added separately to 5 ml 10% F127 (w/v), followed by stirring until the organic solvent evaporated. Post-mixed donor and acceptor InFroMs were made by combining donor solution and acceptor InFroM solutions in equal volume and this solution was incubated overnight before FRET analysis. FRET was assessed based on a decrease in BPc donor emission. Donor fluorescence was measured on a fluorometer (Photon Technology International) with 670 nm excitation and 710 nm emission. A constant amount of BPc donor was used in all samples, with the acceptor ZnBNc added as indicated.

### Molar ratio quantifications

*F127 quantification*. Cobalt thiocyanate was prepared by dissolving 0.3 g cobalt nitrate hexahydrate and 1.2 g ammonium thiocyanate in 3 ml water. 100 μl of cobalt thiocyanate solution, 40 μl F127 solution in the concentration range of 0–7.5 wt% (more concentrated F127 solutions were diluted to fit that range), 200 μl ethyl acetate and 80 μl ethanol were then combined. The mixture was vortexed gently and centrifuged at 14,000*g* for 1 min. The blue supernatant was removed and the blue pellet was washed using ethyl acetate several (∼5) times until the supernatant became colourless. The pellet was then dissolved in 1 ml acetone to measure the absorbance at 623 nm. Drug concentration was quantified by up to four independent methods: (1) freeze dry method: drug-loaded micelles aqueous solution was freeze dried to remove the water and re-suspended in organic solvent (DCM). Absorbance was measured for the quantification of drug. (2) solvent method: a small amount (∼10 μl) of drug loaded micelles in aqueous solution was added to a water miscible organic solvent (ethanol, ∼1 ml) to dissociate the micelles and release the drug. Then absorbance was measured for the quantification of drug. (3) NMR method: the drug loaded micelles were prepared using the same routine preparation method but using D_2_O and NMR spectra were gathered on a Varian Inova-500 MHz or Bruker 600 MHz. VK1 was quantified by the three methods above. (4) HPLC method (for CsA and CTX). A total of 10 μl of InFroMs were diluted in 190 μl DMSO and then centrifuged (5,000*g* for 1 min) and the supernatant was subjected HPLC analysis with a Waters Alliance 2790 instrument. The mobile phase was acetonitrile and the stationary phase was 0.1% trifluoroacetic acid in water. The detection wavelength was 204 nm (for CsA) and 231 nm (for CTX). Molar ratios for clinical formulations were taken from publicly available product monographs. For clinical formulations containing them, ethanol and oil were included in the molar ratio calculations.

### Induced frozen micelle yield as a function of salt concentration

For Cyclosporine A (CsA), 10 mg CsA was dissolved in 1 ml DCM and added to 10 ml of 10% (w/v) F127 solution containing 0, 1, 2 or 3 M NaCl. After stirring for 3 h, the solution was subjected to centrifugal filtration at 0 °C (for 0 and 1 M NaCl) or −10 °C (for 2 and 3 M NaCl) until ∼200 μl of solution was retained or the volume of the retentate was unchanged, and corresponding salt solutions were added back to the concentrate and the washing procedure was performed three times. The retentates were passed through a 0.45 μm syringe filter and HPLC was used to quantify the CsA concentration. To quantify the F127 removal percentage as a function of salt concentration at different temperatures, the filtrates were saved and the cobalt thiocyanate method was used for detection as described above. For T-undec and CTX, 10 mg drug was dissolved in 100 μl DCM and added in 1 ml 10% (w/v) F127 aqueous solution with 0, 1, 2, 3 and 4 M NaCl, followed by stirring for 3 h until the DCM evaporated completely. The solution was subjected to centrifugation at 5,000*g* for 10 min. The pellet was dissolved in 1 ml ethanol, and the absorbance at 240 nm (for T-undec) and 230 nm (for CTX) was measured to quantify unincorporated drugs.

### Co-loading effects

For CTX, 10 mg CTX with different mass ratios of coenzyme Q10 (CTX:CoQ=10:0; 10:0.5; 10:1; 10:2; mg:mg) were dissolved in 100 μl DCM and added to 1 ml 10% (w/v) F127 aqueous solution with 3.5 M NaCl followed by stirring for 5 h and the solutions became clear. The solutions were then diluted 1 in 15 in water at room temperature. At different time points, solutions were subjected to centrifugation at 5,000*g* for 5 min and the supernatant was discarded and 1 ml water was added back to rinse the pellet and the spin process was repeated. After discarding the supernatant, CTX in the pellet was dissolved in 1 ml ethanol and absorbance was measured to quantify the amount of precipitated drug. 3 mg of docetaxel (DTX) or paclitaxel (PTX) with different mass ratio of co-loader (CoQ or Vitamin E) were dissolved in 100 μl DCM and added to 1 ml 10% (w/v) F127 aqueous solution with 4 M NaCl followed by stirring for 5 h. Afterwards, the solutions were diluted 1 in 15 in water, and incubated at room temperature for 1.5 h Solutions were subjected to centrifugation at 5,000*g* for 5 min. The supernatant was discarded and 1 ml water was used to rinse the white pellet and the spin process was repeated. The drug in the pellet was dissolved in 1 ml ethanol and absorbance was measured to quantify the amount of aggregated drug.

### Animal studies and human blood testing

Animal experiments were performed in accordance with the University at Buffalo Institutional Animal Care and Use Committee. Blood was obtained from adult human volunteers following informed consent. Human blood collection was in accordance with the University at Buffalo Institutional Review Board.

*Vitamin K1 (VK1)*. For functional studies, a warfarin sodium (Ark Pharm Inc. #AK-48919) aqueous solution was prepared at a concentration of 16 mg l^−1^. Six-week-old female ICR mice (Harlan) were given free drinking access to warfarin sodium for 24 h before VK1 ss-InFroM intravenous injection. Mice (*n*=6) were injected intravenously with a VK1 ss-InFroM dose at 0,1,2, or 5 mg kg^−1^. The remaining group used as controls without feeding warfarin or any injections. The INR values of the mice were read out by Coagucheck XS system (Roche) 24 h after VK1 administration. For haemolysis, fresh human erythrocytes were collected in citrate from 4 healthy human volunteers. An erythrocyte suspension was obtained by centrifugation at 420*g* for 10 min and isotonic phosphate-buffered saline (PBS) was added. Centrifugation was conducted again at 3,000*g* for 10 min. The supernatant was discarded and the washing process was repeated. Then the stock solution was suspended in PBS at a cell density of ∼8 × 10^9^ cells per ml. 100 mg ml^−1^ VK1 ss-InFroMs was prepared as described above and for a Tween-80 formulation, 200 mg VK1 was dissolved directly in 500 μl Tween-80, and was subsequently diluted 1 in 4 in PBS. VK1 was mixed with 20 μl human erythrocyte stock suspension (with a final volume of 1 ml). A PBS control and a 100% haemolysis samples were made by adding 20 μl human erythrocyte stock suspension into 980 μl PBS or distilled water respectively. Solutions were incubated at 37 °C for 1 h, followed by centrifugation at 2,000*g* for 5 min. The supernatants were transferred to 200 μl DCM, shaken for 5 min for extraction to remove absorbance interference by VK1, followed by subsequent centrifugation at 2,000*g* for 5 min. Haemolysis percentages were determined by absorbance measurements of the water phase at 540 nm.

*Cyclosporine A (CsA)*. An immunosuppression assay was adapted from a previously reported method^43^. Glutaraldehyde was used for fixing sheep red blood cells (SRBCs) to ELISA plates. SRBCs (Innovative Research, Inc. # IR1-020ND) containing 5 × 10^6^ cells in 0.1 ml PBS were added to each well of a flat bottom 96-well ELISA microtiter plates (Thermo Scientific # 267312) and the plates were incubated overnight at 4 °C. 20 μl of 1.8% glutaraldehyde (AMRESCO, # 0904C089) was gently added to wells containing SRBCs and the plate was incubated for 3 min at 25 °C. The final concentration of the glutaraldehyde was 0.3%. SRBCs were washed with saline three times by centrifugation before use. Mice were injected intraperitoneally with 5 × 10^8^ SRBCs in 0.5 ml saline. Four out of five groups of mice (*n*=5) were injected and the remaining group was used as a negative control. CsA ss-InFroMs were injected intravenously 2 days before and 2 days after the single intraperitoneal SRBC injection, with CsA doses of 0, 1, 3 and 10 mg kg^−1^ per injection. Blood was collected 10 days after the last injection of CsA. ELISA plates were washed 4 times with 200 μl PBS and nonspecific binding sites were blocked with 1% skim milk in PBS for 1.5 h. Dilution of serum started with 1:50 in triplicate to the plates and the plate was incubated for 1.5 h at 37 °C. The secondary antibody, HRP-conjugated anti mouse IgM (Santa Cruz Biotechnology, # SC-2064, was then added and incubated for another hr at 37 °C. TMB liquid substrate (Sigma # T0440) was added to each well and the plate incubated for another 30 minutes at 25 °C before the extent of colour formation in each well was determined by measuring absorbance at 450 nm with a TECAN Safire 2 plate reader. Titers were defined as the reciprocal dilution of serum providing an absorbance of >0.2.

*Testosterone undecanoate (T-undec)*. For tracing studies, 2 mg BPc and 100 mgT-undec were used for preparing T-undec ss-InFroMs as described above and and ss-InFroMs were concentrated to 60 mg ml^−1^ T-undec. An oil formulation containing the same amount of BPc was made by dissolving 1.2 mg BPc and 60 mg T-undec in 1 ml castor oil. An amount of 100 μl of ss-InFroMs or castor oil formulation were injected intravenously via tail vein and mice were killed 2 min after injection. Organs were harvested for fluorescence imaging using an IVIS Lumina II system and organs and serum were collected for biodistribution quantification of BPc. Organs were homogenized in 2 ml chloroform and subsequently centrifuged at 3,000*g* for 3 min. Serum was extracted by chloroform overnight. Then absorbance of the supernatants was measured at 700 nm for organs or the organic solvent phase for serum. For functional studies, 5-week-old castrated CD-1 male mice and uncastrated control CD-1 mice were obtained directly from Charles River and were kept for 2 months before assessing testosterone levels by ELISA (Cayman Chemical # 582701) as per manufacturer protocol. ss-InFroMs were then administered with subcutaneous administration and testosterone was monitored as indicated.

*Cabazitaxel (CTX)*. For tumour studies, 5 × 10^6^ MIA PaCa-2 cells (ATCC) were injected at the right groin of female nude mice (Jackson labs) and treatment began when the tumour volumes reached 100 mm^3^. CTX ss-InFroMs were prepared as described above. A Tween-80 formulation similar to a commercial formulation was prepared by dissolving 60 mg CTX in 1,560 mg (1.5 ml) Tween-80, followed by vortexing until the drug was completely dissolved. Before injection, 4.5 ml of 13% (wt/wt) ethanol aqueous solution was added to make a 10 mg ml^−1^ CTX solution, which was diluted further in saline to the required concentration before injection. CTX was administered intravenously injected via tail-vein as indicated. Tumour size was monitored three times per week and mouse weight was measured daily. Mice were killed when the tumour size reached 10 times the original volume. For haemolysis, human erythrocyte measurements were similar to VK1 but without the organic solvent extraction step since there was no significant absorbance crosstalk with haemoglobin. For measurement of complement activation, freshly collected human plasma was centrifuged at 3,000*g* for 10 min. 15 μl of plasma was mixed with 5 μl CTX with concentrations as indicated and were incubated at 37 °C for 30 min. Complement reactions were stopped with 980 μl specimen diluent provided by the supplier. Scb5–9 amounts were quantified by an ELISA kit (Quidel # A020) as per manufacturer instructions.

### Data availability

The raw data used to generate the figures and tables in this manuscript are available from the corresponding author upon request.

## Additional information

**How to cite this article:** Zhang, Y. *et al*. Therapeutic surfactant-stripped frozen micelles. *Nat. Commun.* 7:11649 doi: 10.1038/ncomms11649 (2016).

## Supplementary Material

Supplementary Information Supplementary Figures 1-21 and Supplementary Table 1

## Figures and Tables

**Table 1:** Properties of ss-InFroMs formed from diverse biologically active cargo.

**Compound**	**Drug:F127 molar ratio**	**Drug Concentration (mg ml^−1^)**	**Size (nm)**	PDI*	**Log P**†	**[NaCl](M)**
α-Tocopherol	17	39	86	0.26	8.8	2
Cabazitaxel	8	41	62	0.10	3.7	3.5
Coenzyme Q10	30	43	82	0.28	9.9	4
Cholecalciferal	29	62	45	0.16	8.0	2
Cyclosporine A	15	7	165	0.34	4.1	3
Ergocalciferol	12	25	112	0.31	7.8	2
Ivermectin	45	80	39	0.03	4.4	0
Retinal palmitate	14	33	114	0.25	10.1	2
Squalene	44	80	81	0.28	8.6	2
Teststorone undecanoate	10	60	112	0.19	6.7	4
Vitamin K1	51	150	74	0.25	8.5	0

Values represent the mean from three independent preparations.

^*^Polydispersity index.

^†^Calculated with the ALOGPS 2.1 algorithm.
