# Supplementary material for: Therapeutic surfactant-stripped frozen micelles
Source: Nat Commun. 2016 May 19;7:11649. doi: 10.1038/ncomms11649 (PMC4874033; doi:10.1038/ncomms11649)
Supplement: Supplementary Information — Supplementary Figures 1-21 and Supplementary Table 1 [file ncomms11649-s1.pdf]

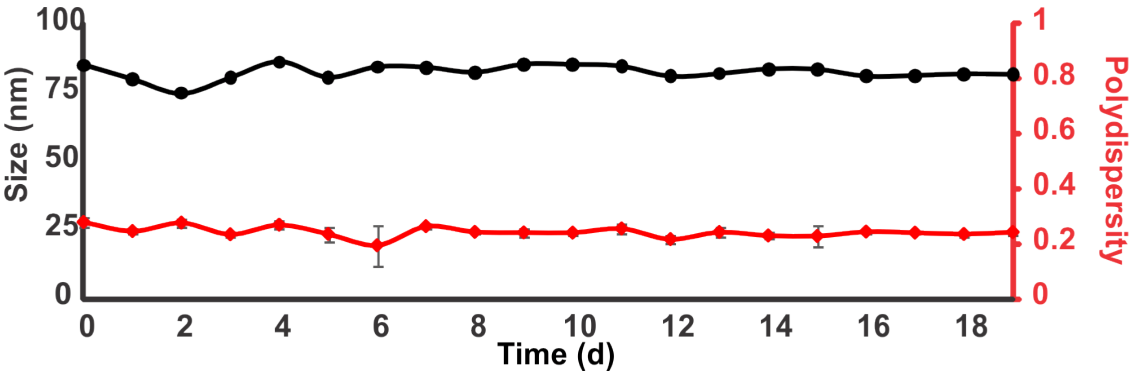

Supplementary Figure 1: Size stability of VK1 ss-InFroMs during storage at 4 °C.

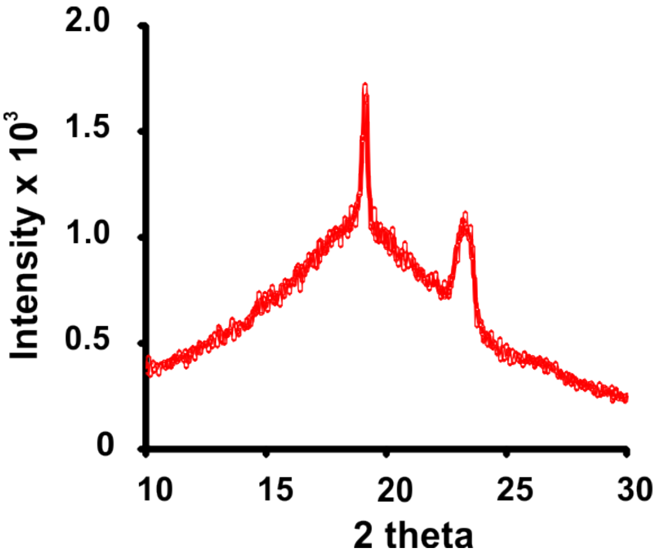

Supplementary Figure 2: X-ray diffraction of freeze dried VK1 ss-InFroMs. The two peaks at 18 and 24 degrees are from F127.

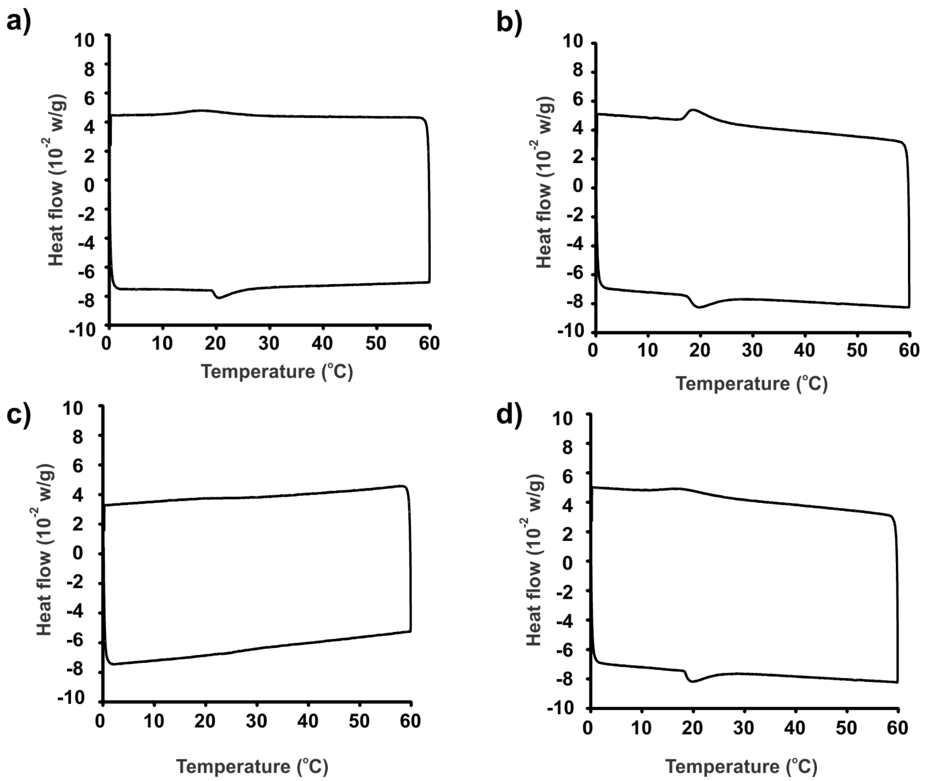

Supplementary Figure 3: Differential scanning calorimetry curves of **a)** VK1 InFroMs (pre wash), **b)** F127 micelles with no drugs loaded, **c)** VK1 ss-InFroMs (post wash) and **d)** VK1 ss-InFroMs diluted back into 10% pristine F127 aqueous solution. Data are representative of 3 independent trials.

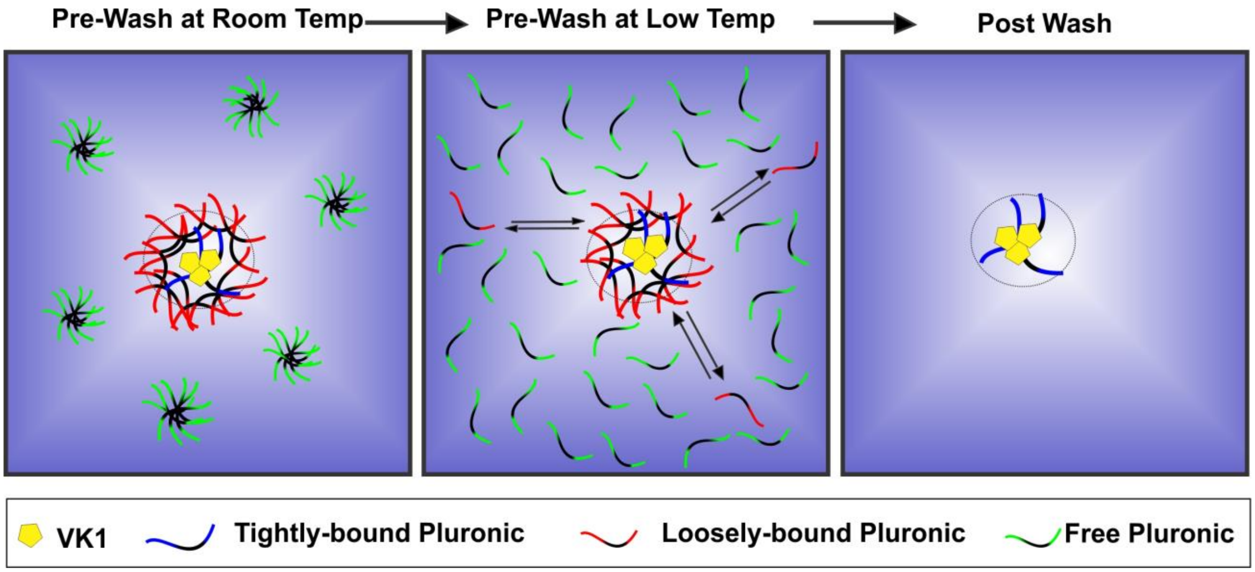

Supplementary Figure 4: Schematic representation of ss-InFroM generation. F127 PPO block are black while PEO of tightly bound, loosely bound and free Pluronic are in blue, red and green, respectively. Hydrophobic cargo is shown in yellow.

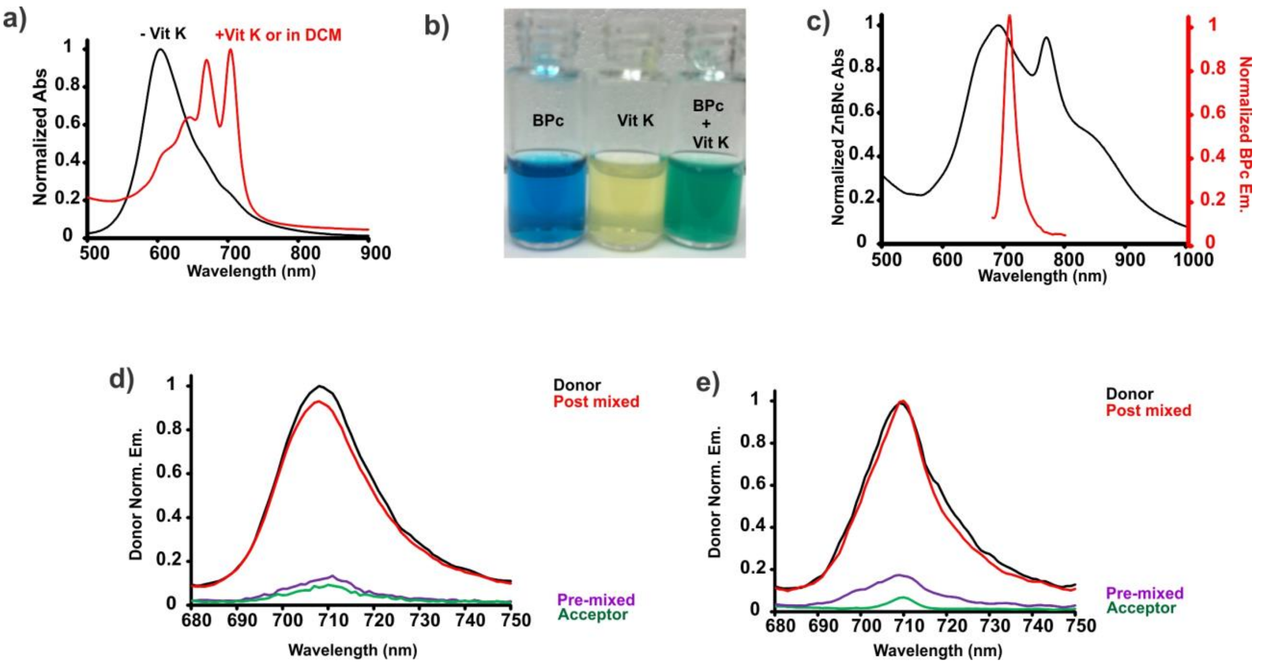

Supplementary Figure 5: Forster resonance energy transfer (FRET) assay to monitor inter-micelle exchange. **a)** Absorbance spectrum of BPc-loaded micelles with and without VK1 co-loaded and in DCM solution. **b)** Photograph of BPc, VK1, and pre mixed of both loaded micelles. Inclusion of hydrophobic VK1 modulated BPc properties, accompanied by a change of color. **c)** Overlap of spectra for FRET. Mix of donor and acceptor micelles still remained significant fluorescence for pre **(d)** and post **(e)** removal of surfactant after 2 weeks. Cargos are locked in frozen micelles without inter-micellar exchange. Data are representatives of 3 independent trials.

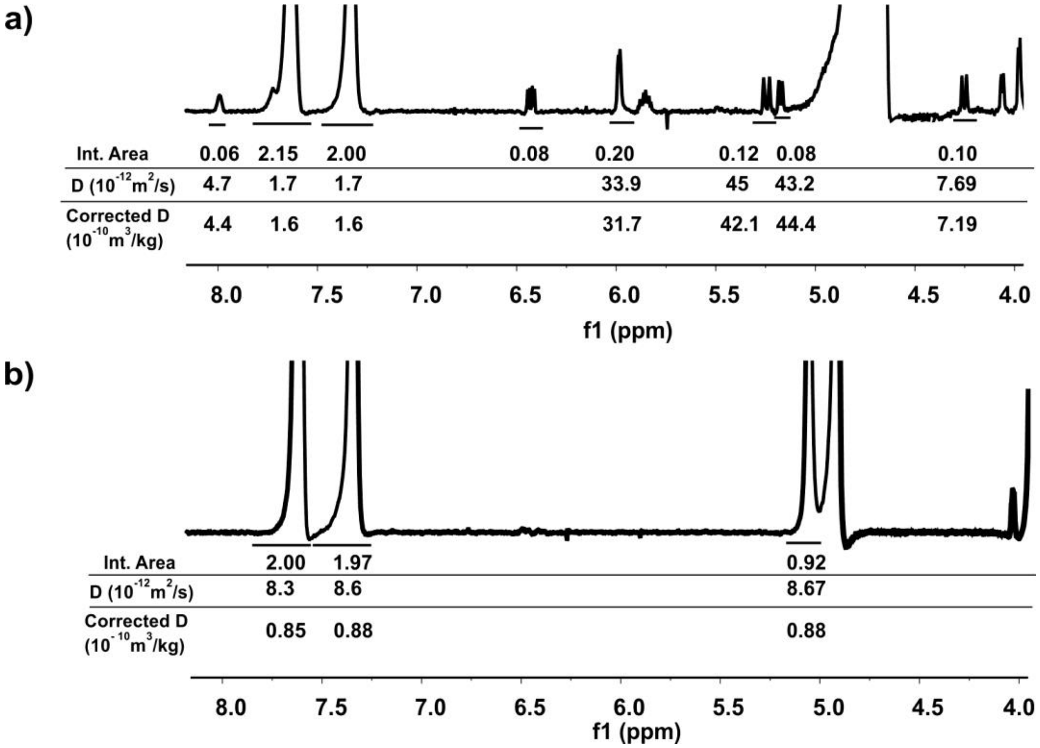

Supplementary Figure 6: Diffusion ordered NMR spectroscopy before and after removal of excess F127. 1-D NMR spectrum of **a)** pre and **b)** post washed VK1 InFroMs. Inset table numerates integration areas of peaks and diffusion coefficients of corresponding hydrogens with and without viscosity correction. Difference of diffusion coefficients (after viscosity correction by Einstein stokes equation) of pre and post wash samples might be due to removal of interactions of VK1 exerted by free surfactant (the sizes of pre and post sample are almost the same). Data representatives of 3 independent trials.

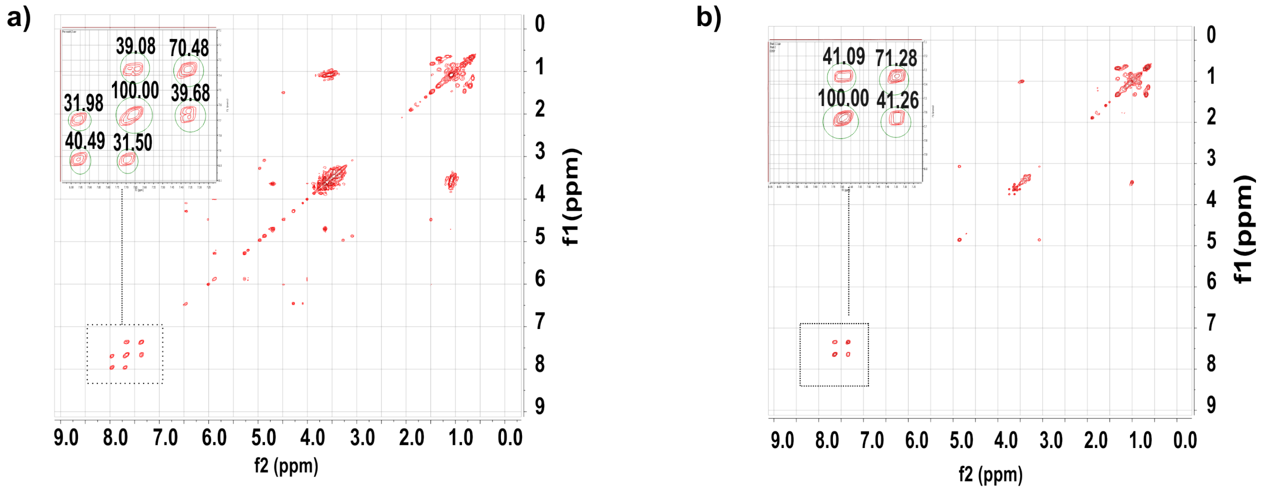

Supplementary Figure 7: Correlation spectroscopy (COSY) NMR for **a)** pre and **b)** post washed VK1 InFroMs. Data representatives of 3 independent trials.

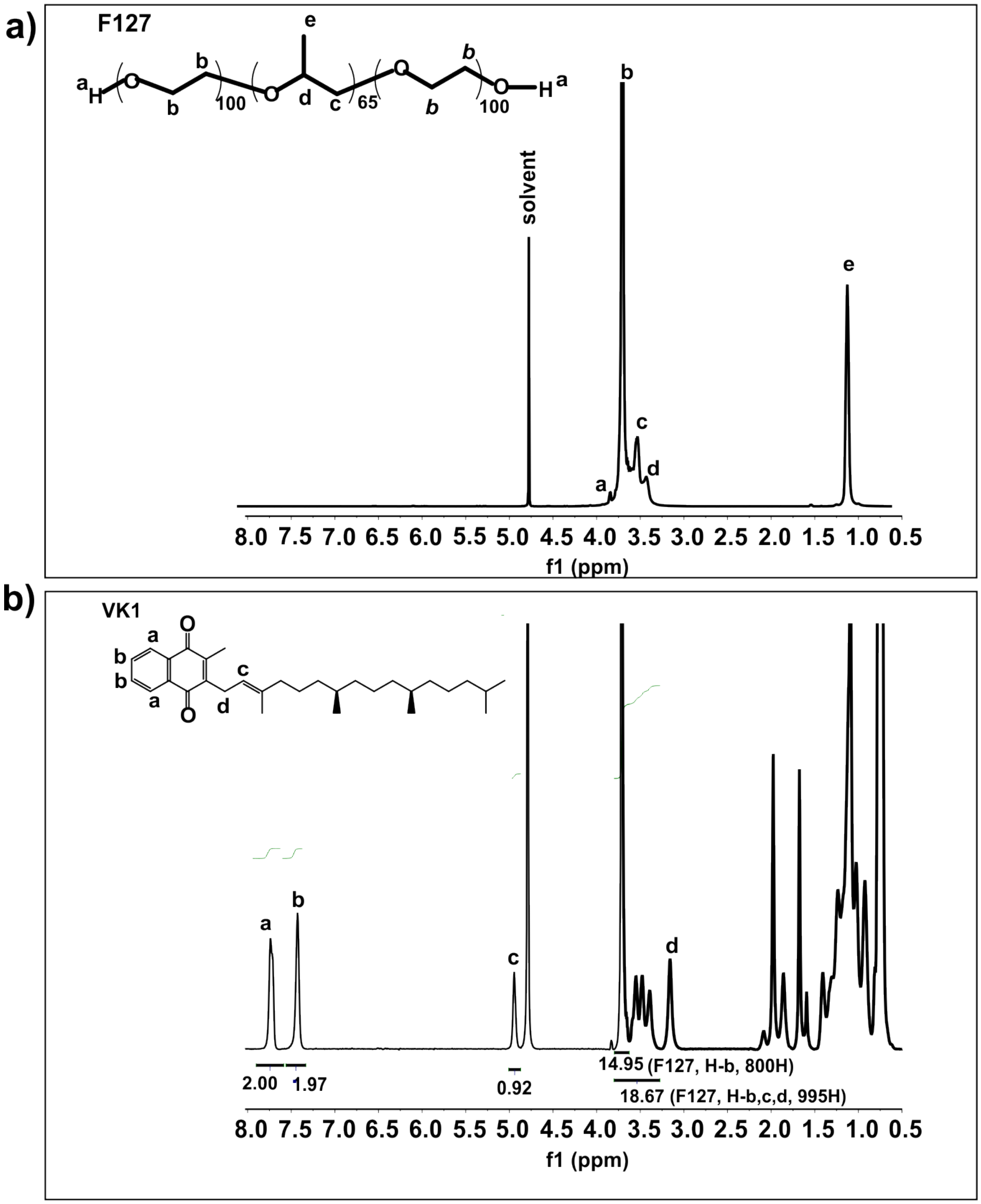

Supplementary Figure 8: NMR spectrum for molar ratio quantification a) NMR spectrum of Pluronic F127 with inset of its chemical structure**. b)** NMR spectrum of VK1 ss-InFroMs (post wash) with inset of chemical structure of VK1. (Solvent used for NMR is deuterium oxide and measurements were done in triplicate)

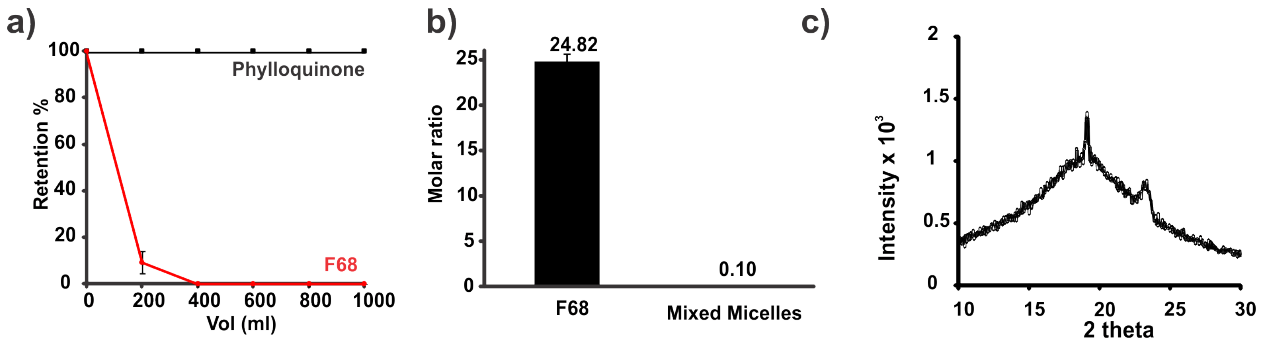

Supplementary Figure 9: Characterization of VK1 F68 ss-InFroMs. **a)** Retention percentage of VK1 (Phylloquinone) and Pluronic F68 during surfactant stripping process. **b)** Molar ratio of VK1 F68 ss-InFroMs and mixed micelles. **c)** X-ray diffraction of freeze dried VK1 F68 ss-InFroMs (two peaks at 18 and 24 degree are from F127).

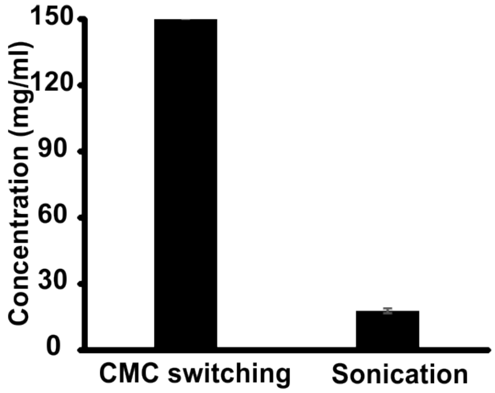

Supplementary Figure 10: Enhanced drug solubilization using CMC switching. VK1 was formed using either CMC switching and surfactant stripping compared or by directly dissolving VK1 powder into a F127 solution yielding an equivalent concentration of VK1 and F127, followed by sonication for 2 hours. Data show mean +/- std. dev. for n=3.

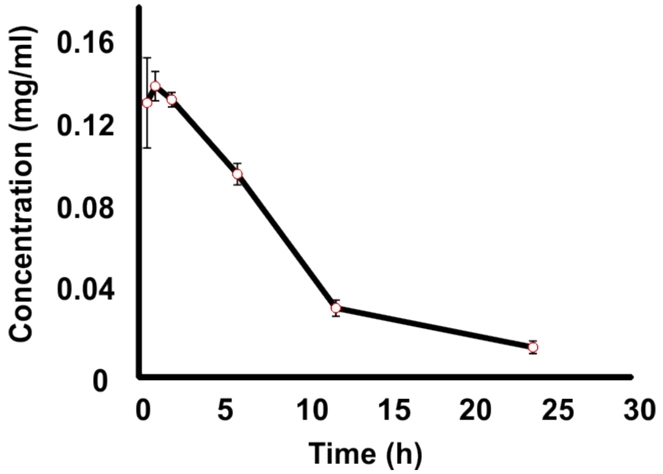

Supplementary Figure 11: Blood clearance of VK1 ss-InFroMs following intravenous administration in mice. Data show mean +/- std. dev. for n=3 mice.

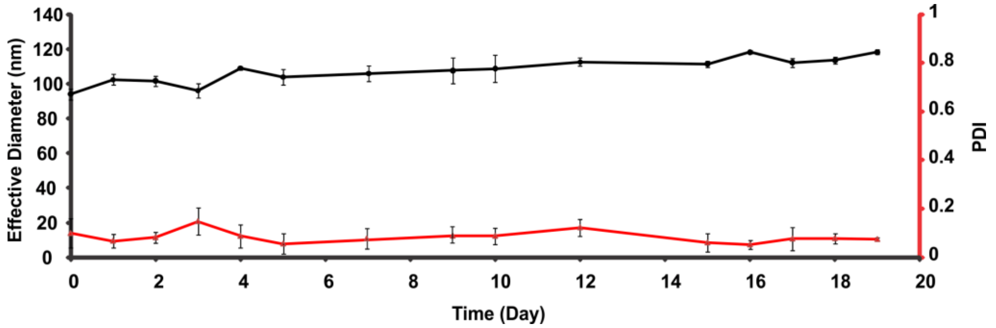

Supplementary Figure 12: Size stability of CsA ss-InFroMs during storage at -20 °C in 3 M NaCl. Data show mean +/- std. dev. for n=3.

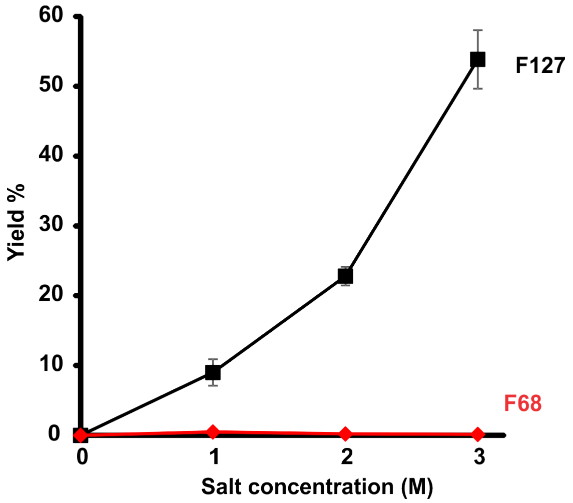

Supplementary Figure 13: Yield of CsA ss-InFroMs using Pluronic F127 (black) and F68 (red) carriers at different NaCl concentrations. Data show mean +/- std. dev. for n=3.

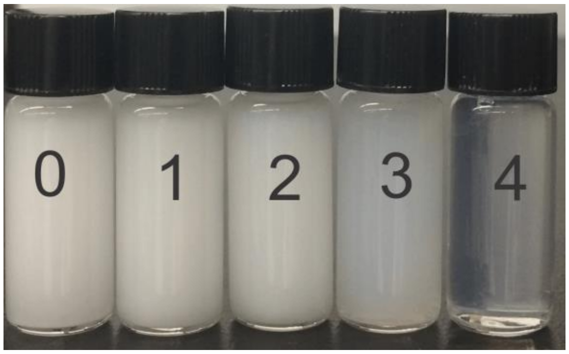

Supplementary Figure 14: Photograph of T-undec. InFroMs in hypertonic saline. T-undec InFroMs were prepared in 10 % F127 (w/v) with different NaCl concentrations (0, 1, 2, 3, 4 M as indicated by the numbers in the photo). Results are representative of 3 independent trials.

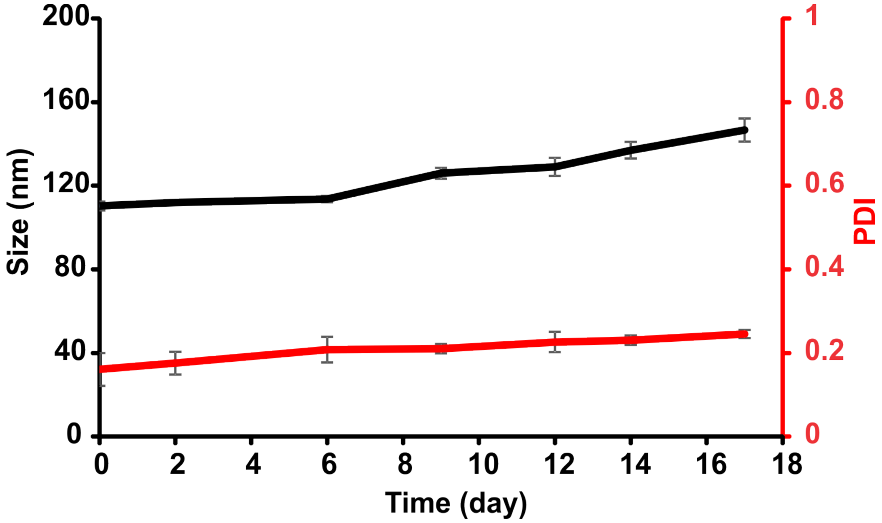

Supplementary Figure 15: Size stability of T-undec ss-InFroMs (in 4 M NaCl) during storage at -20 °C. Data show mean +/- std. dev. for n=3.

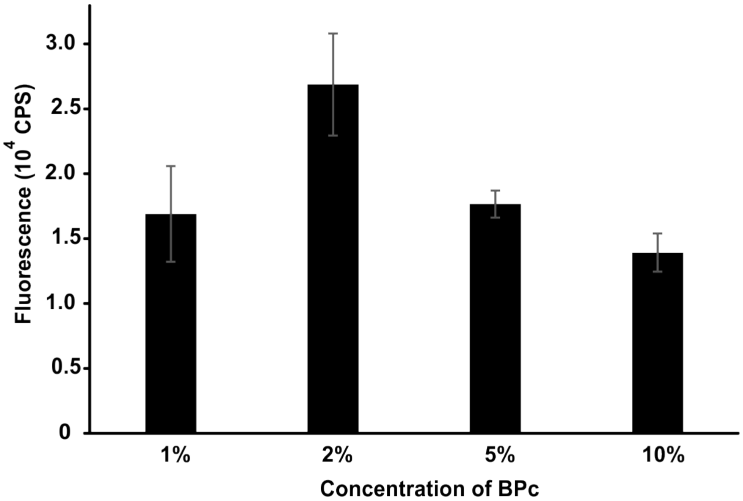

Supplementary Figure 16: Fluorescence of different mass concentrations of BPc in T-undec InFroMs. The sample with 2% BPc is the most fluorescent due to maximized concentration and minimal fluorescence self-quenching.

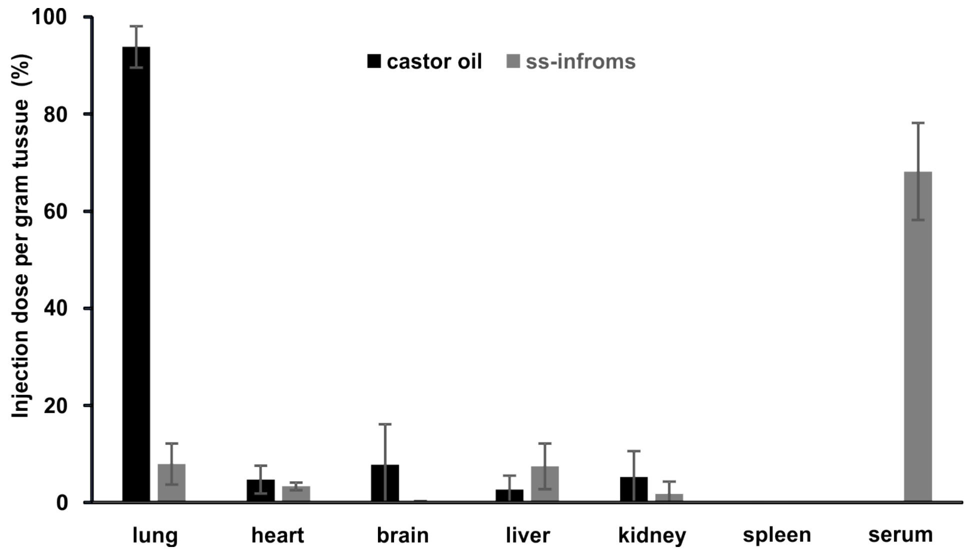

Supplementary Figure 17: Biodistribution of BPc-doped T-undec formulations (in castor oil or ss-InFroM form) 2 minutes following intravenous injection in mice. Data show mean +/- s.d. for n=3 mice per group.

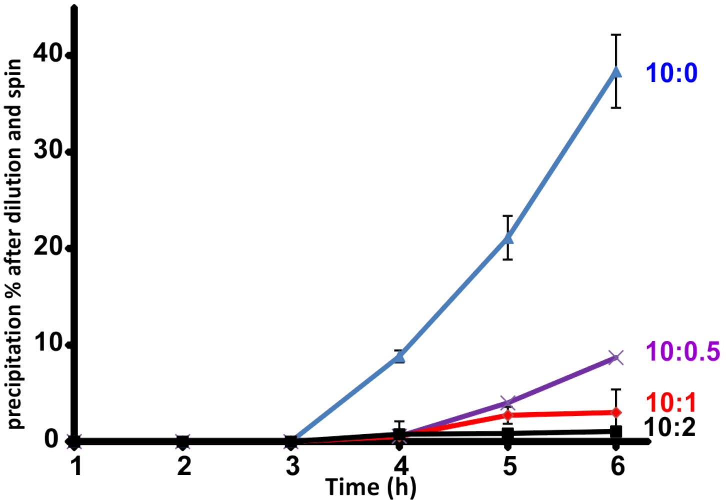

Supplementary Figure 18: Aggregation kinetics of co-loaded CTX InFroMs**.** CTX InFroMs co-loaded with varying mass ratios of CTX to CoQ (10:0, 10:0.5, 10:1, 10:2) were formed in 3.5 M NaCl and diluted 1 in 15 in water prior to assessing aggregation over time. Data show mean +/- std. dev. for n=3.

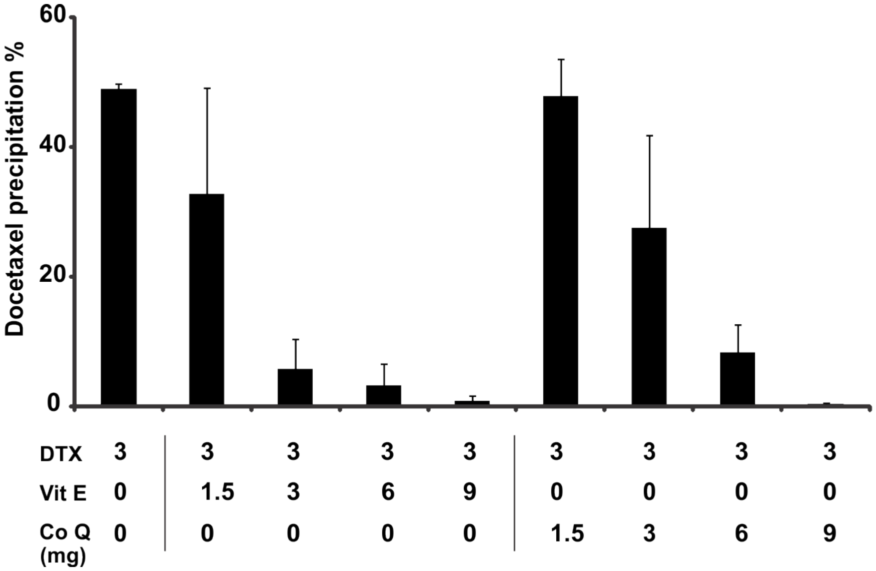

Supplementary Figure 19: Co-loading effect of docetaxel (DTX) InFroMs (pre wash) with Vitamin E or Coenzyme Q10 as indicated, following dilution in water. Data show mean +/- std. dev. for n=3.

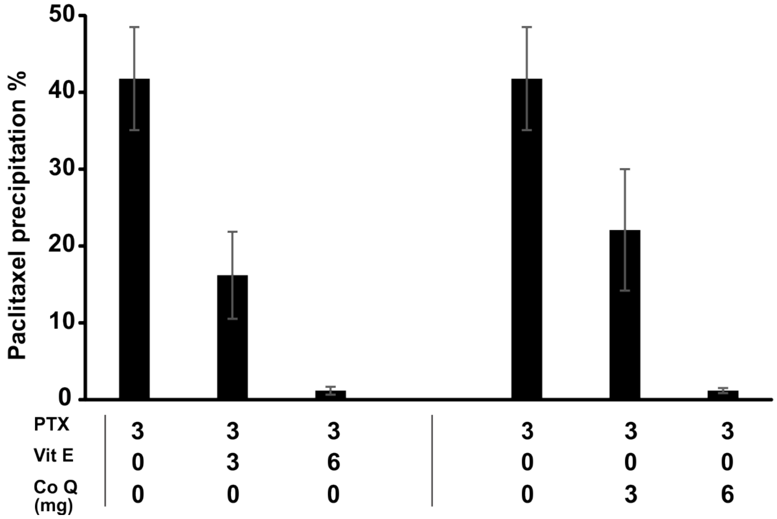

Supplementary Figure 20: Co-loading effect of paclitaxel (PTX) InFroMs (pre wash) with Vitamin E or Coenzyme Q10 as indicated, following dilution in water. Data show mean +/- std. dev. for n=3.

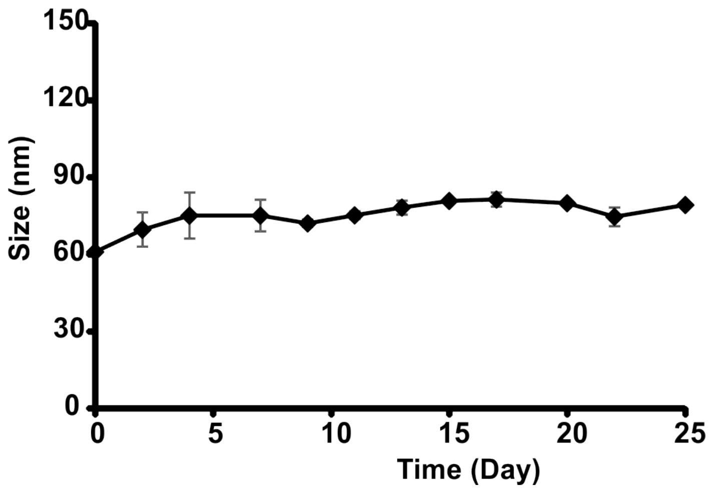

Supplementary Figure 21: Size stability of CTX ss-InFroMs (in 3.5 M NaCl) during storage at -20 °C. Data show mean +/- std. dev. for n=3.

Supplementary Table 1: Physical parameters of Pluronic F127 and F68

Pluronic type	N of PEO	N of PPO	% of PPO	HLB	MW (g/mol)	C1 (wt%)	C2 (wt%)
F127	100	65	25	22	12600	0.7 [a]	18[c]
F68	76	29	16	29	8400	10-11 [b]	58[c]

N: number of blocks per polymer
HLB: Hydrophilic-lipophilic balance
MW: Molecular weight
C1: critical micellization concentration at 25 °C
C2: transition concentration from isotropic micelle form to mesophase at 25 °C

Source:
[a] Lin and Alexandridis, J. Phys. Chem. B, 106, 10834-10844, 2002.
[b] Clark et al, J Colloid Interface Sci, 354, 144-151, 2011.
[c] Wanka et al, Macromolecules, 27, 4145-4159, 1994.
